# SSCA-Net: Simultaneous Self- and Channel-Attention Neural Network for Multiscale Structure-Preserving Vessel Segmentation

**DOI:** 10.1155/2021/6622253

**Published:** 2021-03-30

**Authors:** Jiajia Ni, Jianhuang Wu, Jing Tong, Mingqiang Wei, Zhengming Chen

**Affiliations:** ^1^Shenzhen Institutes of Advanced Technology, Chinese Academy of Sciences, China; ^2^College of Internet of Things Engineering, Hohai University Changzhou, China; ^3^Nanjing University of Aeronautics and Astronautics, China

## Abstract

Vessel segmentation is a fundamental, yet not well-solved problem in medical image analysis, due to the complicated geometrical and topological structures of human vessels. Unlike existing rule- and conventional learning-based techniques, which hardly capture the location of tiny vessel structures and perceive their global spatial structures, we propose *Simultaneous Self- and Channel-attention Neural Network* (termed SSCA-Net) to solve the *multiscale structure-preserving vessel segmentation* (MSVS) problem. SSCA-Net differs from the conventional neural networks in modeling image global contexts, showing more power to understand the global semantic information by both *self- and channel-attention* (SCA) mechanism and offering high performance on segmenting vessels with multiscale structures (e.g., DSC: 96.21% and MIoU: 92.70% on the intracranial vessel dataset). Specifically, the SCA module is designed and embedded in the feature decoding stage to learn SCA features at different layers, in which the self-attention is used to obtain the position information of the feature itself, and the channel attention is designed to guide the shallow features to obtain global feature information. To evaluate the effectiveness of our SSCA-Net, we compare it with several state-of-the-art methods on three well-known vessel segmentation benchmark datasets. Qualitative and quantitative results demonstrate clear improvements of our method over the state-of-the-art in terms of preserving vessel details and global spatial structures.

## 1. Introduction

Vessel segmentation is aimed at automatically or semiautomatically detecting the boundaries (consisting of pixels) of blood vessels within 2D or 3D medical images such as *computed tomography* (CT) or *magnetic resonance angiography* (MRA) images. As one of the most challenging tasks in *medical image segmentation* (MIS), vessel segmentation can deliver significant information about the shapes and volumes of vessels, which are critical to the diagnosis and treatment of vascular diseases [1, 2].

**Figure 1:**
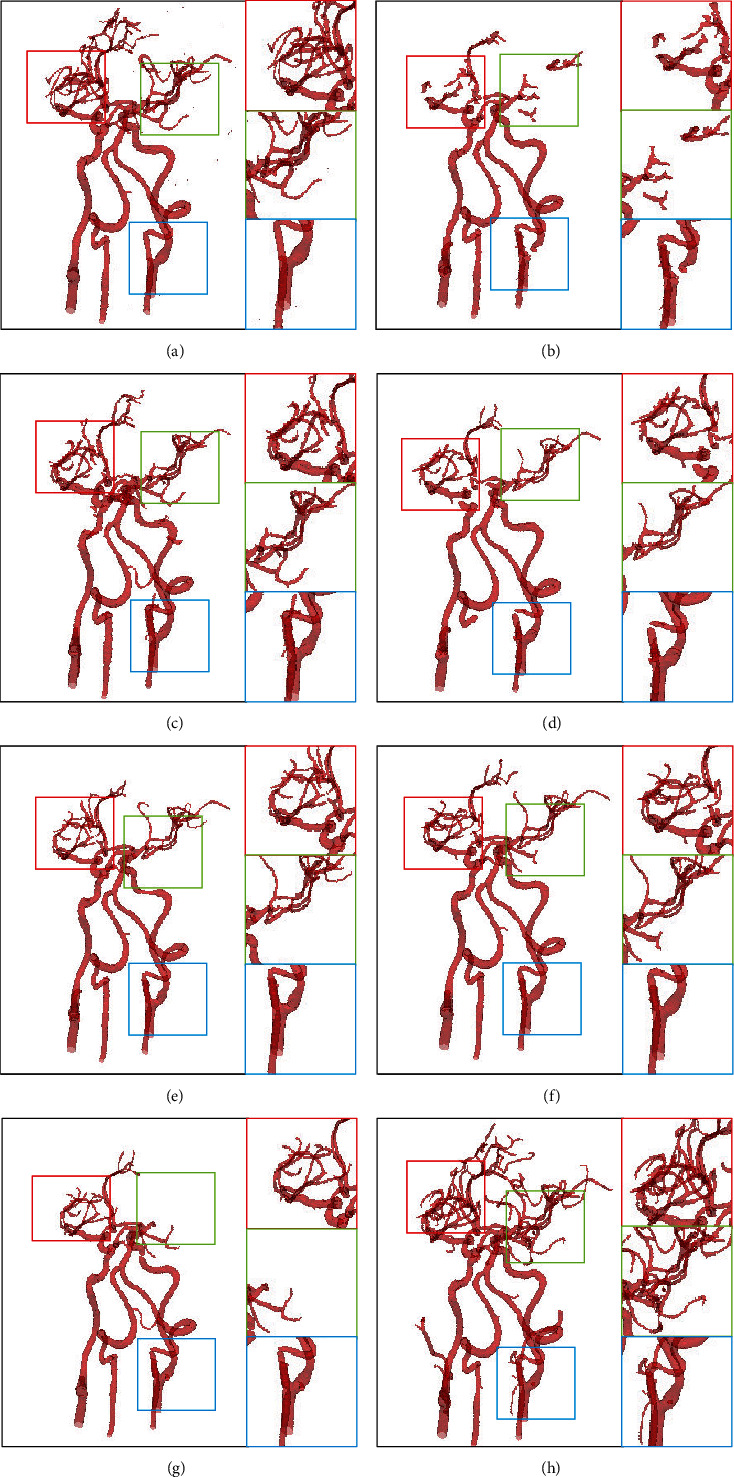
Medical image segmentation results tested in the dataset of the intracranial artery. (a) Ground truth and the segmentation results of (b) DeepASPP [10], (c) DeepLab V3+ [9], (d) ENet, (e) FCN8s [3], (f) RefineNet [11], (g) U-Net [6], and (h) ours, respectively. Our SSCA-Net can perform the segmentation of intracranial arteries effectively while preserving multiscale structures of vessels, especially the tinny-scale structures.

The most successful type of models for vessel segmentation is deep learning-based techniques, especially *convolutional neural network-* (CNN-) based frameworks, which have shown to be a powerful and robust tool in segmenting homogeneous areas of medical images [3–11], as shown in Figure 1. Although those methods had achieved state-of-the-art performance for many segmentation tasks, it is also faced with the following problems: they usually use a large number of feature information, while these features may have different importance for the segmentation task. Intuitively, maintaining lots of feature maps or complex network structures can boost the segmentation performance. However, doing that is not optimal to both reduce network overfitting and improve the segmentation accuracy. Besides, due to the increase in the depth of CNN, it is easy to cause the network to lose some spatial feature information and channel feature information. To address these problems, people began to use the attention mechanism.

Recent studies have validated that the attention-based global features are important for semantic segmentation. For instance, the pyramid attention network [12] exploits the impact of global contextual information on semantic segmentation and uses global attention upsampling to replace the bilinearly upsampling. However, these methods utilize consecutive pooling and stride convolutional operations to capture global feature information. As known, this kind of operation can lead to the loss of location and spatial information.

We argue that the attention mechanism is helpful for vessel segmentation and assume that taking a more comprehensive use of attention would boost the segmentation performance. In this paper, we propose a novel module named *self-attention and channel-attention* (SCA), which can be used to connect low-level and high-level features, compared to the standard U-Net, which uses simple skip connections to connect low-level and high-level features. Our proposed SCA block could capture wider and deeper semantic features by infusing the attention mechanism. Additionally, we also use *squeeze and excitation pyramid pooling* (SEPP) [13], which can extract enriched feature representations in the same multiscale pooling operations. It can better increase the resilience and robustness of the network. Furthermore, to streamline the network structure, we replace ResNet [14] block with the *ReLU Feature Unit* (RFU). In summary, the RFU block is proposed to reduce model parameters and optimize network structure, followed by the SEPP block for further context information with multiscale pooling operations. Integrating the RFU block and the SEPP block with the backbone encoder-decoder structure and use the SCA module as skip connection structure, we develop an end-to-end vessel segmentation neural network named SSCA-Net. The main contributions of this work are summarized as follows:

1. We propose an SCA block to get more abstract spatial and channel features and preserve more multiscale spatial information
2. We propose novel building networks including a multiscale spatial and feature attention module, a novel multiscale feature fuse module, and a simple feature extraction block that decreasing model parameters
3. We apply the proposed method to three different datasets, namely, intracranial blood vessels, retinal vessel data, and leg vessel data. Results show that the proposed method outperforms the state-of-the-art methods in these different tasks

The paper is organized as follows: Section 2 discusses related work. The architectures of the proposed SSCA-Net models are presented in Section 3.  Section 4 explains experiments, results, discussion, and ablation study. The conclusion is discussed in Section 5.

## 2. Related Work

### 2.1. Traditional Deep Learning

Deep learning-based semantic segmentation methods can be roughly divided into two categories: FCN-based and U-Net-based. The FCN-based methods are characterized by the direct use of high-level semantic segmentation, which removes the last two full-connect layers to classify each pixel. Due to solely using high-level features, FCN-based methods perform not well and even lead to less accuracy on some datasets. To address this problem, a variety of improvements are proposed, e.g., combining FCN with graphical models like *Markov Random Fields* (MRFs) [15] and *Conditional Random Fields* (CRFs) [16, 17] to refine the segmentation prediction. Furthermore, the U-Net-based methods have been proposed, which can be characterized by using a skip connect to combine low-level and high-level features to predict the segmentation. It has become a popular neural network architecture and has shown promising results on different medical image segmentation tasks [7, 11, 18].

### 2.2. Context Aggregation

In recent years, various methods have explored contextual information by many researchers, which are more complicated than the U-Net, for example, the DeepLab series [9, 19, 20]. The DeepLab method introduced the *atrous convolution* and *atrous spatial pyramid pooling* (ASPP) [9] network structure. The latest DeepLab V3+ [9] extended DeepLab by adding a decoder module and using depth-wise separable convolution (Xception [21]) for better performance and efficiency. PSPNet [22] adopted the pyramid pooling module to partition the feature map into different scale regions. Yu et al. [23] developed a Context Prior to distinguish the intraclass and interclass context clearly. Lin et al. proposed a multipath refinement network, which contains residual convolution unit, multiresolution fusion, and chained residual pooling. Yang et al. [10] proposed the *densely connected atrous spatial pyramid pooling* (DenseASPP), which connects a set of *atrous convolutional* layers densely. Furthermore, to improve the resilience of the network, the pyramid structure of the space is applied to semantic segmentation [24, 25].

### 2.3. Attention Model

The attention mechanism was first successfully applied in natural language processing tasks, and then, it was well extended to solve image processing tasks. Zhao et al. [26] proposed the pointwise spatial attention network to guide contextual information collection. The *squeeze-and-excitation* (SE) networks [27] adopted a channel-wise relationship attention mechanism to enhance the representational power of the network. Woo et al. [28] proposed *Convolutional Block Attention Module* (CBAM) for feed-forward convolutional neural networks. CCNet [29] utilized the self-attention mechanism to obtain contextual information. Zhong et al. [30] proposed a novel squeeze-and-attention network architecture for obtaining an enhanced pixel-wise prediction. *Bottleneck attention module* (BAM) [31] used a simple yet effective attention module, which infers an attention map along channel and spatial. Ni et al. [13] proposed a spatial and channel-based attention-based convolutional neural network (GC-Net) to segment medical image data. Our SSCA-Net network is different from the methods mentioned above. The contextual information is aggregated by both self-attention and channel-attention modules.

## 3. Methodology

**Figure 2:**
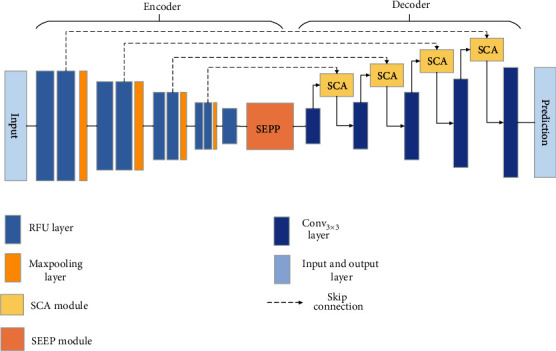
Illustration of the proposed SSCA-Net. We use multiple times of *ReLU Feature Unit* (RFU) module as feature encoder. Then, the feature maps are fed into a feature decoder module. It contains a *self- and channel-attention* (SCA) block and a *squeeze and excitation pyramid pooling* (SEPP) block. Moreover, we adopt skip connection to connect the low-level feature maps and high-level feature maps.

We propose a new framework that provides multiple modules over which information from the feature encoder module and decoder module is assimilated using a generic building block, the SSCA-Net, as shown in Figure 2. We begin by describing the SCA module in Section 3.1 followed by a detailed description of each SSCA-Net block in Section 3.2.

### 3.1. Self- and Channel-Attention (SCA) Module

**Figure 3:**
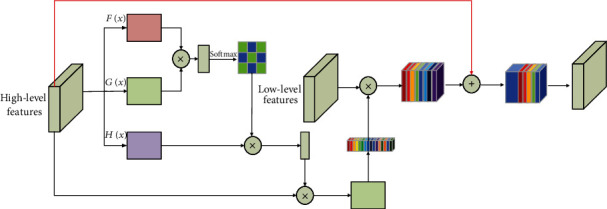
The designed *self- and channel-attention* (SCA) module for aggregating high-level features and low-level features. “⊗” denotes spatial element-wise multiplication, and “⊕” denotes element-wise sum. The red lines represent the upsampling operators.

As noted previously, we aim to exploit attention features for prediction with long-range residual connections. Hence, we propose an SCA module, as shown in Figure 3. In the classic image segmentation network model, multiple convolutional layers are used to preserve the local neighborhood information of the image. However, the modeling of long-range dependence of images by convolutional neural networks is inefficient. Therefore, we adapt the *nonlocal* (NL) model [32] to introduce self-attention to the image semantic segmentation framework.

Additionally, the upsampling portion of the image generation network typically uses a deconvolution network. Besides, convolution kernel sizes and step sizes can cause deconvolution operations to generate checkerboard artifacts. To avoid the checkerboard effect, we use bilinear interpolation as an upsampling operation.

The image features from the previously hidden layer *x* ∈ *R*^*H*×*W*^ are first transformed into two feature spaces *F*(*x*) and *G*(*x*) to calculate the attention.

(1) $$\begin{array}{c} F\left(x\right)=W_{f}x,\\ \;G\left(x\right)=W_{G}x,\\ \beta_{i,j}=\frac{\exp\left(s_{i,j}\right)}{\sum_{i=1}^{N}\exp\left(s_{i,j}\right)},\\ s_{i,j}=F\left(x_{i}\;\right)G\left(x_{j}\right), \end{array}$$

where *β*_*i*,*j*_ indicates the extent to which the model attends to the *i*^th^ location when synthesizing the *j*^th^ region. Then, the output of the attention layer is *β* = (*β*_1_, *β*_2_, *β*_3_ ⋯ ⋯*β*_*j*_ ⋯ ⋯*β*_*N*_), where

(2) $$\begin{array}{c} \beta_{j}=\overset{N}{\underset{i=1}{\sum }}\beta_{j.i}H\left(x_{i}\right),\\ H\left(x_{i}\right)=W_{H}x_{i}. \end{array}$$

In the above formulation, *W*_*f*_ ∈ *R*^*H*×*H*^, *W*_*G*_ ∈ *R*^*H*×*H*^, *W*_*H*_ ∈ *R*^*H*×*H*^ are the learned weight matrices, which are implemented as 1 × 1 convolution. Finally, the features are again element-wise multiplication operation with the feature *x*_*i*_. In short, the operation is computed as follows:

(3) $$\begin{array}{c} \mu_{i}=\beta_{j}x_{i}. \end{array}$$

Also, the feature map should be aggregated in each channel. To this end, we take global average pooling on the attention layer feature map *μ*_*i*_ and produce a channel vector *X*_*c*_ ∈ *R*^*c*×1×1^. Then,  *X*_*c*_ and *X*_low_ perform the element-wise multiplication operation and produce a multiplication vector *X*_*m*_ ∈ *R*^*C*×*H*×*w*^. This last obtained feature vector *X*_*m*_ is combined with a bilinearly interpolated feature vector *X*_hig_. Therefore, the final output is given by

(4) $$\begin{array}{c} y_{i}=\left(\text{aver}\left(\mu_{i}\right)⊗X_{\text{low}}\right)+\text{upsame}\left(X_{\text{hig}}\right), \end{array}$$

where aver is the global average pooling and upsame is the upsampling operation. ⊗ denotes element-wise multiplication.  *X*_low_ and *X*_hig_  represent low-level feature maps and high-level feature maps, respectively.

### 3.2. SSCA-Net Block

The architecture of SSCA-Net is illustrated in Figure 2. Our architecture is generic, and each SSCA-Net block can be easily modified to accept an arbitrary number of feature maps with arbitrary resolutions and depths.

#### 3.2.1. ReLU Feature Unit (RFU)

The first part of each SSCA-Net block consists of the RFU that is mainly for feature learning. We do not use ResNet block in this task, since the medical image is not included in the category of the pretrained model. And it can also prevent overfitting and reduce both network parameters and training time. The RFU can reduce the training time and accelerate network convergence.

Mathematically, the RFU block can be formulated as

(5) $$\begin{array}{c} \text{RFU}=\text{ReLU}\left(\text{BN}\left({\text{Conv}}_{3\times 3}\left(x\right)\right)\right), \end{array}$$

where ReLU is an activation function and BN denotes the *batch normalization*. Conv_3×3_ is the convolution operation with the kernel size of 3.

Therefore, the feature encoder module network structure can be expressed as follows:

(6) $$\begin{array}{c} {\text{layer}}_{0}=\text{RFU}\left(x\right),\\ {\text{layer}}_{1}=\text{maxpooling}\left({\text{layer}}_{0}\right),\\ {\text{layer}}_{i}=\text{RFU}\left({\text{layer}}_{i-1}\right), \end{array}$$

where *i* is the number of downsampling, e.g., it takes “4” in the intracranial artery and the leg bone artery, and it takes “3” on the retinal vessel set.

#### 3.2.2. Squeeze and Excitation Pyramid Pooling (SEPP)

**Figure 4:**
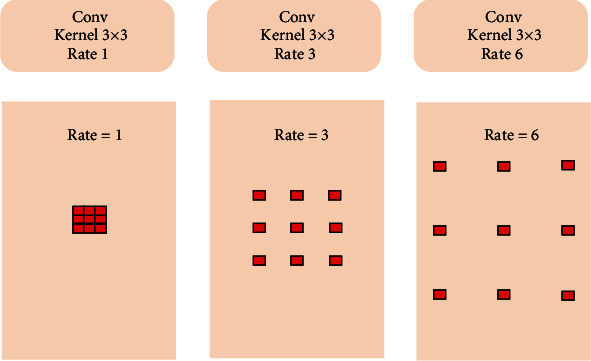
The illustrations of *atrous convolution*.

**Figure 5:**
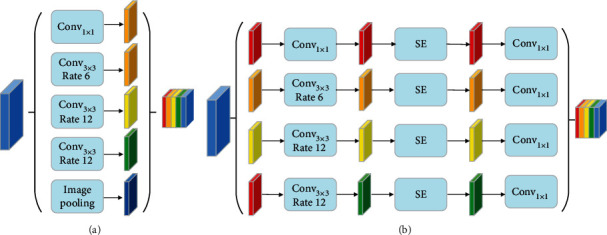
Illustration of (a) atrous spatial pyramid pooling (ASPP) and (b) squeeze and excitation pyramid pooling (SEPP).

In semantic segmentation, multiple convolutions and pooling operations may lead to the reduction of the receptive field of the network and the loss of information features in different layers. To overcome this limitation, *atrous convolution* (see Figure 4) and *spatial pyramid* model (see Figure 5) are adopted for semantic segmentation. Due to pyramid pooling, it can counteract the shrunken receptive field by assembling multiscale context. For example, *pyramid scene parsing* (PSP) [22] performs spatial pooling at several grid scales and demonstrates outstanding performance on several semantic segmentation benchmarks. In the classic ASPP network, there are four parallel *atrous convolutions* with different atrous rates in the feature coding stage. Different from [9], we combine the SE operation into the residual block in ASPP to readjust the dynamic channel characteristics.

In this case, the SEPP module is also different from GC-Net [13]. Here, the SEPP module (see Figure 5) has four cascaded branches with the gradual increment of the number of *atrous convolution* and SE network structure. Since a large receptive field can acquire much contextual information, we present 4 dilated convolutions whose dilation scales are 1, 6, 12, and 12 in SEPP. In each branch, we apply 1 × 1 convolution for rectified linear activation after each *atrous convolution* and SE network.

Mathematically, the SEPP block can be formulated as

(7) $$\begin{array}{c} \text{SEPP}=\text{Cat}\left[\begin{array}{c} {\text{Conv}}_{1\times 1,}\left(\text{SE}\left\{{\text{Conv}}_{1\times 1}\left(x\right)\right\}\right),\\ {\text{Conv}}_{1\times 1,}\left(\text{SE}\left\{{\text{Conv}}_{3\times 3,d_{y}}\left(x\right)\right\}\right) \end{array}\right]\;y\in \left[6,12,12\right],\\ \text{SE}=\text{sigmoid}\left(\text{ReLU}\left(\text{aver}\left(x\right)\right)\right), \end{array}$$

where Conv_1×1_ denotes the 1 × 1 convolutions and Conv_3×3,*d*_*x*__ denotes the dilation convolutions with the kernel size of 3 × 3 and the dilation scale is *x*. Cat(∗)  is a concatenating operation, and *x* is the input feature map. Sigmoid is the full connection with the *sigmoid* activation function. ReLU is the full connection with the ReLU activation function.

Therefore, the feature decoder module network structure can be expressed as follows:

(8) $$\begin{array}{c} {\text{layer}}_{0}=\text{SCA}\left(\text{SEPP}\left({\text{Conv}}_{i+1}\right),{\text{Conv}}_{i}\right),\\ {\text{layer}}_{1}=\text{BN}\left({\text{Conv}}_{1\times 1}\left({\text{layer}}_{0}\right)\right),\\ {\text{layer}}_{x}=\text{SCA}\left({\text{layer}}_{x-1},{\text{Conv}}_{i-x+1}\right), \end{array}$$

where *x* is the number of upsampling and  *i* is the number of downsampling, which should be equal to *x*. We use BN to reduce internal covariate shift [33] and Conv_1×1_ to reduce feature dimensions and complexity of training. SCA(∗) and SEPP(∗) represent the SCA module and the SEPP module, respectively.

## 4. Experiments

### 4.1. Experimental Settings

To show the effectiveness of our approach, we carry out comprehensive experiments on three datasets: intracranial blood vessel dataset, *Digital Retinal Images for Vessel Extraction* (DRIVE) [34], and leg arteries. The segmentation quality is measured by the *dice similarity coefficient* (DSC) [13], *mean intersection-over-union* (MIoU) score [35], the *sensitivity* (Sen), and the *accuracy* (Acc) [36]. We also introduce the *area under the receiver operation characteristic curve* (AUC) to measure segmentation performance on DRIVE. We apply simple data augmentation during training on the intracranial blood vessel dataset and leg arteries, including affine transformation, rotation, and vertical flip operations. We also performed data augmentation on DRIVE, including gray-scale conversion, standardization, contrast-limited adaptive histogram equalization, and gamma adjustment.

#### 4.1.1. Intracranial Blood Vessel Dataset

We first present our results on the intracranial blood vessel dataset in this work courtesy of a local hospital in Shenzhen, China. The imaging modality of the dataset is *computed tomography angiography* (CTA). There are 4326 CTA images of intracranial blood vessels with a dimension of 512 × 512 in the original dataset. During the training, 20% of images are used as the validation set, while the remainder 80% as the training set. We also use two new patient data as the test data which are not included in the training and validation set.

#### 4.1.2. DRIVE

The second application is retinal vessel detection. The DRIVE dataset has been obtained from a diabetic retinopathy screening program in the Netherlands which contains 40 photographs. These are equally divided into 20 images for training and the other 20 images for testing. Due to the limited amount of data, we use subimages for training. Each 128 × 128 patch is obtained by randomly selecting its center inside the full image.

#### 4.1.3. Leg Arteries

The next application is the leg artery segmentation task. The imaging modality of the leg blood vessel dataset is CTA from a local hospital in Shenzhen, China. There are 6545 CTA images of leg blood vessels with a dimension of 512 × 512 in the original dataset. During the training, 20% of images are used as the validation set, while the remainder 80% as the training set. In addition, we use two new patient data as the test data which are not included in the training and validation set.

#### 4.1.4. Training Details

In the training stage, we use the ADAM [37] optimizer with the initial learning rate of 1*e*–3, *β*1 = 0.5, and *β*2 = 0.999, and the initial rate lr = 1*e*‐3. The initial learning rate is multiplied by  (1 − (epoch − 1/totalepoch)^power^) where the power is set to 0.9. The maximum number of epochs is 200. In this work, the loss function is the same as GC-Net. The implementation is based on the public Keras [38] platform with TensorFlow [39] as backend. The training and testing bed is an Ubuntu 16.04 system.

### 4.2. Test on the Intracranial Blood Vessel Dataset

**Figure 6:**
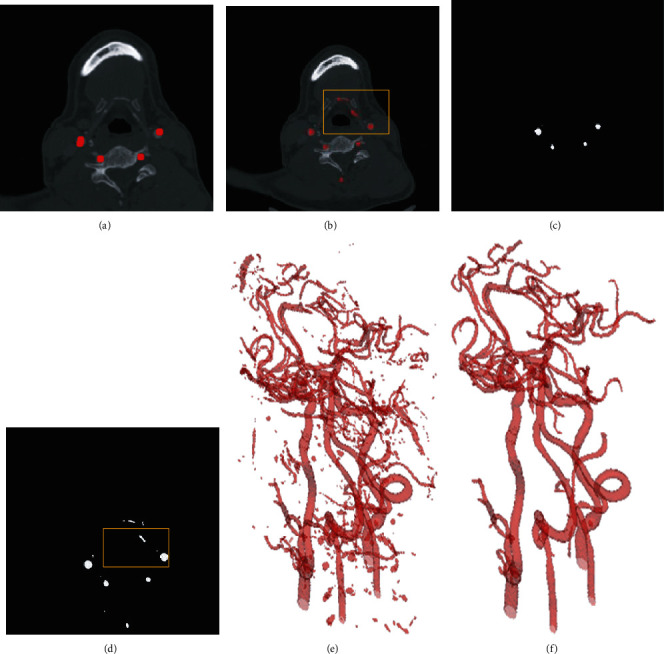
The effectiveness of postprocessing. (a, c) Some nonvessel areas have been removed after postprocessing, compared to the results of (b, d) before postprocessing. (e, f) The 3D results before and after postprocessing, respectively.

The 3D reconstruction of segmented vessels (consisting of 2D CT slices) can validate the segmentation quality by visually demonstrating their spatial information. It can be observed in Figure 6 that there are some noises on the surface as isolated objects, arising from the misclassifications.

It is known that the entire intracranial arteries are interconnected. However, the missegmented noise is not connected to the entire blood vessel. As shown in Figure 6, there are some unconnected noises near each blood vessel. Therefore, we removed some areas or noises, accounting for less than 0.03% of the entire blood vessel.

**Figure 7:**
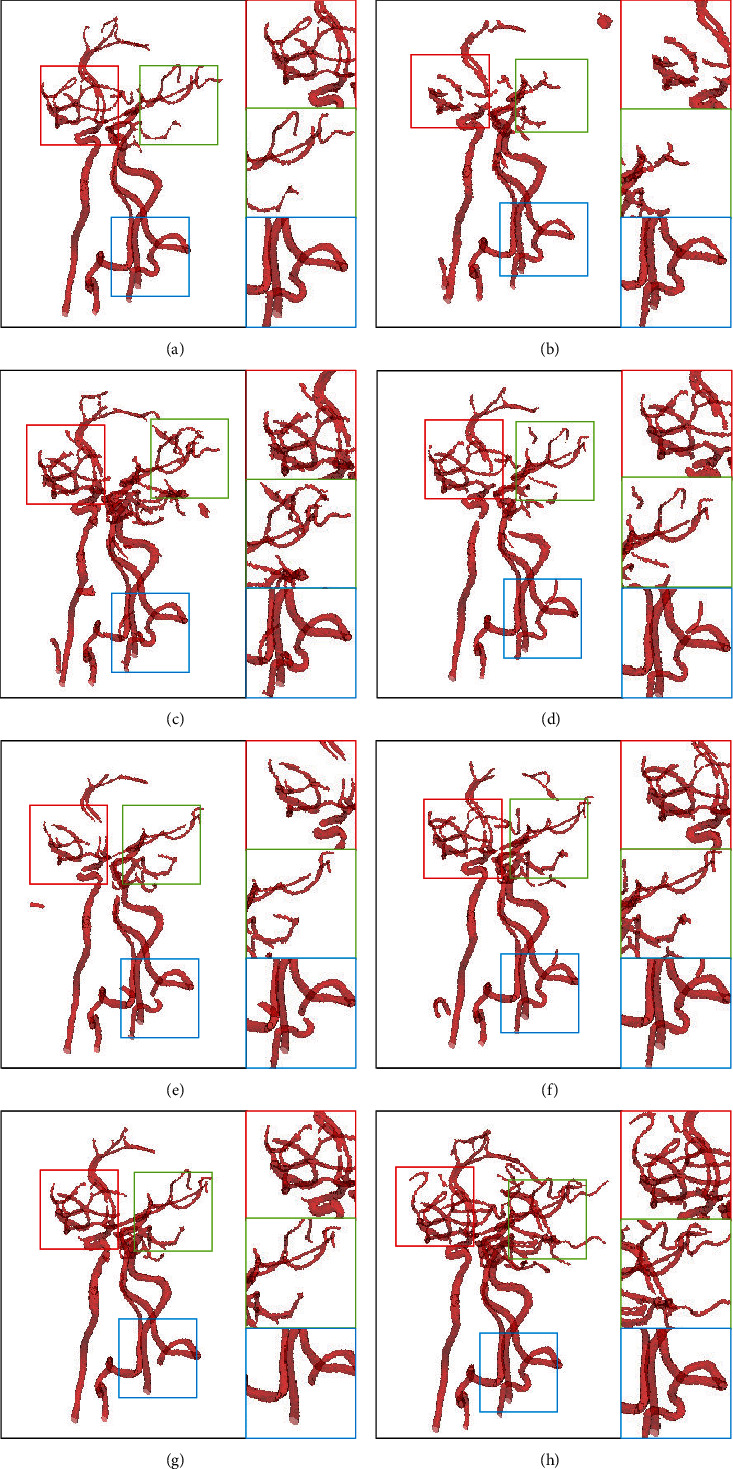
Medical image segmentation results tested in the dataset of the intracranial artery. (a) Ground truth and the segmentation results of (b) DeepASPP [10], (c) DeepLab V3+ [9], (d) ENet, (e) FCN8s [3], (f) RefineNet [11], (g) U-Net [6], and (h) ours, respectively. Our SSCA-Net can perform segmentation of intracranial arteries effectively while preserving more vessel tinny-scale structures.

Postprocessing is not performed to better explain the effect of SSCA-Net, as shown in Figure 7. As pointed out in the yellow circles, some segmented areas are either noise or real vessels. We can see some more small-scale structures produced by our SSCA-Net, in terms of ground truths. After postprocessing, we can see more clearly. This is because the ground truth is manually labeled, and some of the vessels are too small; the marker does not notice. This result also demonstrates that the SSCA-Net can effectively perform semantic segmentation.

Numerical results of our SSCA-Net and the state-of-the-art semantic segmentation solutions on intracranial blood vessel datasets are summarized in Table 1. These results are obtained under the same experimental conditions and the same data pretreatment. The DSC score of segmentation accuracy increased from 76.14% to 96.21%, and the accuracy of MIoU increased from 66.53% to 92.70%. In particular, as we can see in Figures 1 and 7, SSCA-Net produces more multiscale structures than other methods. The reason is that the SCA module and the SEPP module can well preserve the information of medical images.

### 4.3. Test on Retinal Vessel Segmentation

**Figure 8:**
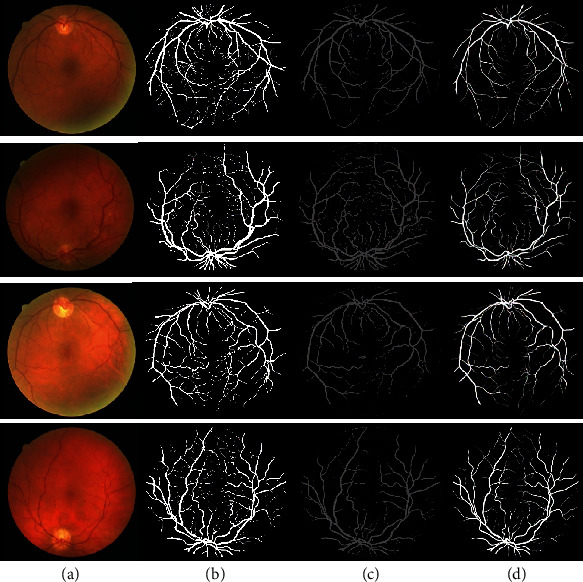
Visualization results on the DRIVE dataset. (a) Test image, (b) ground truth, and results of (c) U-Net and (d) SSCA-Net, respectively.

We have compared the proposed SSCA-Net with CNN-based algorithms [6, 13, 40, 41] and some classical methods [42–46].  Table 2 shows the comparison of our method to those methods. From the comparison, the SSCA-Net achieved 98.20%, 83.52%, and 96.14% in AUC, Sen, and Acc, respectively, which are better than the other methods. Comparing with the CE-Net, the AUC increases from 97.79% to 98.20%, and that the sensitivity score increases from 83.09% to 83.52% while the accuracy increases from 95.45% to 96.14%, which shows that the SSCA-Net is beneficial for retina vessel detection. We show some examples for visual comparisons in Figure 8.

### 4.4. Test on Leg Arteries

**Figure 9:**
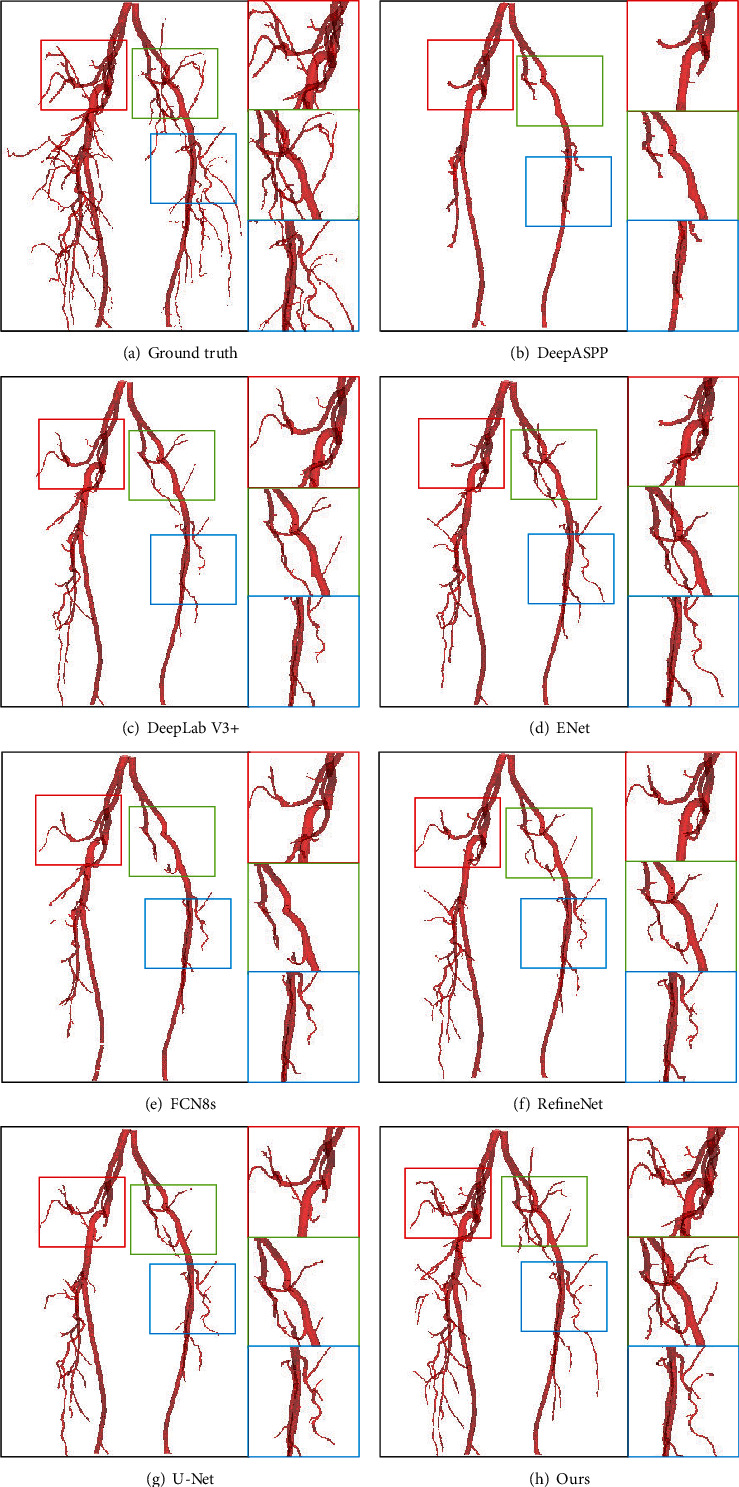
Comparative visualization of 3D results achieved on the test dataset 1 and test dataset 2. Compared to the ground truth, all the state-of-the-art methods (DeepASPP, DeepLab V3+, ENet, FCN8s, RefineNet, and U-Net) miss fine features (e.g., small vessels in the rectangle), whereas the proposed method preserves fine vessels well.

We have compared our SSCA-Net with the state-of-the-art algorithms as shown in Table 3. Our proposed method achieves the performance, which the DSC score is 97.21% and the MIoU score is 94.42%. Comparing with the FCN16s, the DSC score increases from 83.41% to 97.21% by 16.5%, and the MIoU score increases from 65.12% to 94.42%, which shows that the skip connected is beneficial for semantic segmentation. Besides, comparing with U-Net, the DSC score increases from 91.25% to 97.21%, and the MIoU score increases from 76.26% to 94.42%, which shows that the proposed SCA and SEPP blocks are beneficial for leg vessel segmentation as well. We also compared some of the existing excellent methods, and the results show that SSCA-Net can perform blood vessel segmentation more effectively. We show some examples for visual comparisons in Figure 9.

### 4.5. Discussion and Ablation Study

To verify the efficacy of different modules in our method, we conduct the ablation study. We also give several design choices and show their influences on the results.

*Backbone.* The modified U-Net without the pretrained ResNet50 and with the SCA block.

*Backbone+ASPP.* The network with the SCA block but without the SEPP block and replaces it with ASPP.

*ResNet50+SCA.* The network with the pretrained ResNet50 and SCA.

*ResNet50+SCA+SEPP.* The network with the pretrained ResNet50, SCA, and SEPP.

*ResNet50+SCA+ASPP.* The network with the pretrained ResNet50, SCA, and ASPP.

#### 4.5.1. Analysis of Pretrained Weight

**Figure 10:**
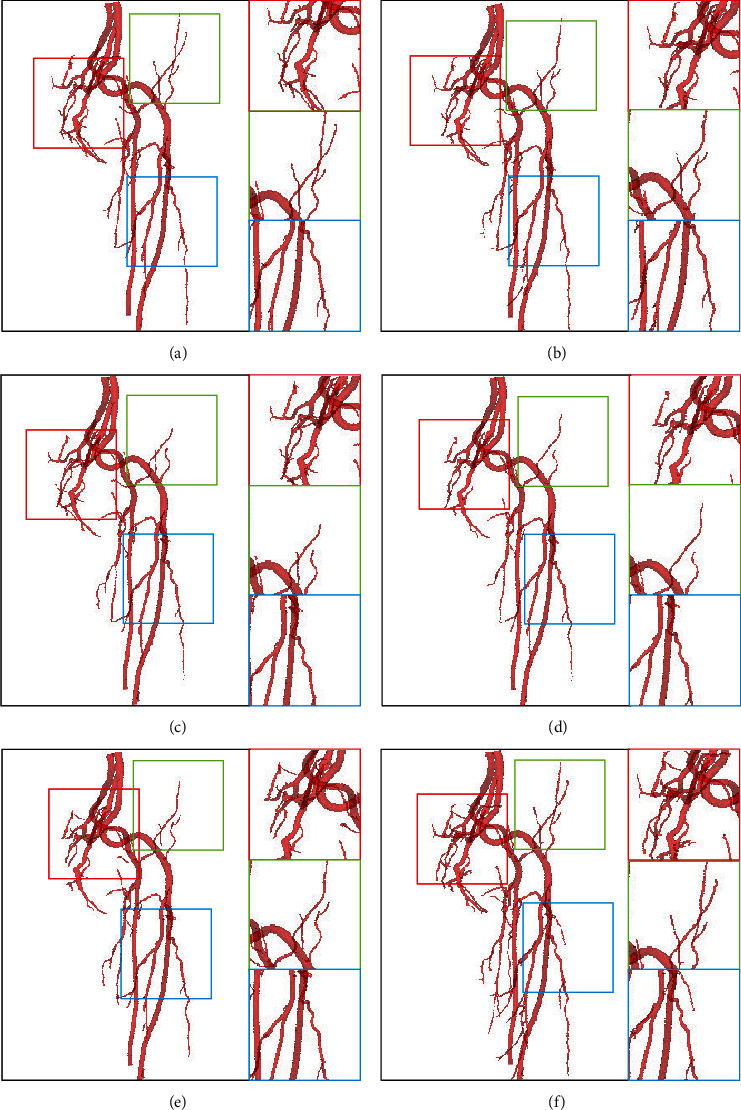
Medical image segmentation results tested in the dataset of leg artery. (a) Backbone, (b) Backbone+ASPP, (c) ResNet50+SCA+ASPP, (d) ResNet50+SCA, (e) ResNet50+SCA+SEPP, and (f) SSCA-Net, respectively. Our SSCA-Net can perform segmentation of intracranial arteries effectively while preserving more vessel details.

**Figure 11:**
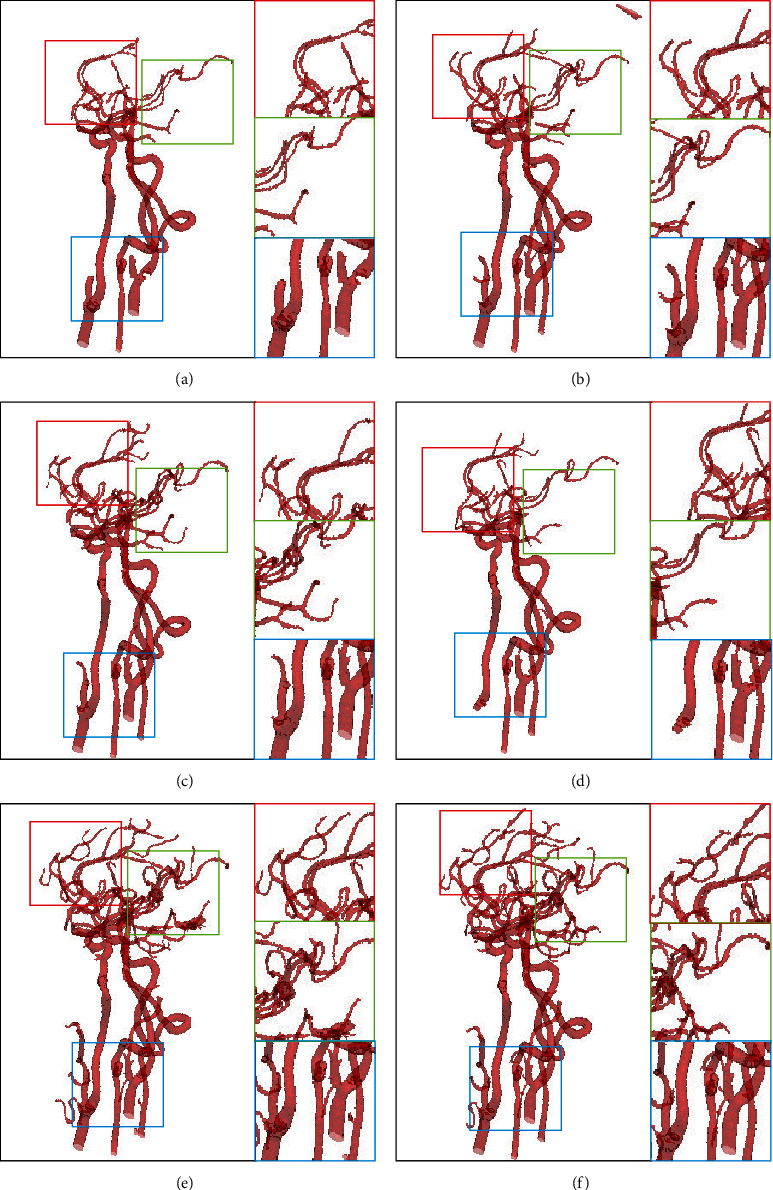
Medical image segmentation results tested in the dataset of intracranial artery. (a) Backbone, (b) Backbone+ASPP, (c) ResNet50+SCA+ASPP, (d) ResNet50+SCA, (e) ResNet50+SCA+SEPP, and (f) SSCA-Net, respectively. Our SSCA-Net can segment intracranial arteries effectively while preserving more vessel details.

Recent work [47] points out that ImageNet pretraining is no better than the original feature encoder in terms of model training accuracy. We do ablation learning on two datasets because the two datasets contain a large amount of data, which can better verify the potential of the network. On the intracranial arterial blood vessel dataset and the leg arterial blood vessel dataset, we can see that ResNet50+SCA+SEPP has increased from 95.79% and 96.78% to 96.21% and 97.21% in DSC and MIoU increased from 91.70% and 93.75% to 92.70% and 94.42%. The results in Figures 10 and 11 and Tables 4 and 5 have demonstrated the effectiveness of without pretraining weights which is not worse than using weights.

#### 4.5.2. Analysis of SEPP and SCA

*(1) SEPP*. In Tables 4 and 5, we validated the effect of incorporating SE into the improved ASPP module. Considering the characteristics of the network, we designed two experiments. One is the use of pretraining weights, and the other is without the use of pretraining weights. The results can be seen on the two arterial blood vessel data. It demonstrates that the receptive field plays a significant role in semantic segmentation. It can be seen in Tables 4 and 5 that the network structure using the SEPP module has improved in both the DSC and MIoU evaluation standards compared to the use of ASPP and networks that do not use similar structures. This is because medical images contain very little information compared to natural images, and it is easy to cause information loss when using a large number of convolution and pooling operations.

*(2) SCA*. Similarly, we apply experiments to verify the effectiveness of the SCA module. In this paper, if the pretraining weights and SCA modules are not used, this network can be regarded as a U-Net network. In Tables 1 and 3, we can see that the SSCA-Net network structure is better than U-Net.

#### 4.5.3. Comparison with GC-Net

Both are our best segmentation model (in Table 1) and SEPP model. We can see that SSCA-Net has a slight decrease compared to GC-Net in the DSC evaluation standard, but it has an improvement in MIoU. These subtle differences are in the range of tolerance, and the reason for this mainly comes from the fine-tuning batch normalization parameters.

#### 4.5.4. Ablation Study

Finally, it has been proved that the proposed algorithm is accurate and robust in medical segmentation from various CT images (see Figures 6–11). The average DSC and MIoU of the proposed method on intracranial blood vessel data were 96.21% and 92.70%, respectively, which are shown in Table 1. On the leg bone artery dataset, the average DSC and MIoU were 97.21% and 94.42%, respectively, which are shown in Table 3. On the retinal vessel dataset, Sen, Acc, and AUC obtained 85.32%, 96.14%, and 98.20%, respectively, on the three evaluation criteria, and the results were better than other methods, which are shown in Table 2.

*(1) Limitations*. We have introduced two new modules to deal with the problem of medical image segmentation from CT images. To some extent, the SSCA-Net network structure can better improve the segmentation accuracy of CT images. But compared to the U-Net network, it requires more parameters and takes a little more time to train the network. In different experiments, we have observed that the more feature information, the better the performance, but in this work, due to the lack of medical data, we conduct experiments on 2D slices. However, to get more segmentation image information, 3D data may be used in the future.

## 5. Conclusion

This paper presents a novel network, called SSCA-Net, for multiscale structure-preserving vessel segmentation. SSCA-Net mainly uses two attention mechanisms to analyze the context information of the entire network. To obtain global contextual information, we introduce the SCA attention module which applies two attention modes to obtain the feature information of the image, the SEPP module is devised to increase the size of the receptive field of the network while learning more features, and design a weighted cross-entropy loss function to make the training process more effective. These operations are beneficial for improving the accuracy of vessel segmentation with multiscale structures. Furthermore, we also experimented with the feature encoder module instead of the ResNet50 pretraining model. This greatly reduces the training time and also reduces the problem of network overfitting. Our method can be applied to different tasks by fine-tuning our model using the new training data and test on three benchmark datasets and is compared with various state-of-the-art methods concerning the DSC, MIoU, and AUC metrics.

## Figures and Tables

**Table 1:** Comparison with the state-of-the-art methods on the intracranial blood vessel training dataset.

Method	DSC (%)	MIoU (%)
U-Net [6]	87.32	86.48
FCN8s [3]	84.23	67.72
FCN16s [3]	76.14	66.53
DenseASPP [10]	84.38	81.80
DeepLab V3+ [9]	90.70	87.83
RefineNet [11]	91.68	76.72
GC-Net [13]	96.35	91.89
SSCA-Net	96.21	92.70

**Table 2:** Performance comparison of the competing methods on retina vessel data using different performance metrics.

Method	Sen (%)	Acc (%)	AUC (%)
Azzopadi et al. [42]	76.55	94.42	96.14
Roychowdhury et al. [43]	72.50	95.20	96.72
Zhao et al. [44]	74.20	95.40	86.20
U-Net [6]	73.44	95.23	97.44
DeepVessel [40]	76.03	95.23	97.52
HED [41]	73.64	94.34	97.23
Li et al. [45]	75.69	95.27	97.38
Melinscak et al. [46]	—	94.66	97.49
CE-Net [36]	83.09	95.45	97.79
GC-Net [13]	78.44	95.51	97.77
SSCA-Net	83.52	96.14	98.20

**Table 3:** Comparisons with state-of-the-arts on leg blood vessel training dataset.

Method	DSC (%)	MIoU (%)
U-Net [6]	91.25	76.26
FCN8s [3]	88.52	80.11
FCN16s [3]	83.41	65.12
RefineNet [11]	95.21	91.85
DeepASPP [10]	88.36	87.02
DeepLab V3+ [9]	92.05	90.57
SSCA-Net	97.21	94.42

**Table 4:** Performance comparisons of context aggregation approach on leg blood vessel data.

Method	DSC (%)	MIoU (%)
ResNet50+SCA+SEPP	96.78	93.75
ResNet50+SCA+ASPP	96.51	93.53
ResNet50+SCA	96.67	93.37
Backbone+ASPP	96.92	94.19
Backbone	96.98	94.07
SSCA-Net	97.21	94.42

**Table 5:** Performance comparisons of context aggregation approach on intracranial blood vessel data.

Method	DSC (%)	MIoU (%)
ResNet50+SCA+SEPP	95.79	91.70
ResNet50+SCA+ASPP	95.72	90.69
ResNet50+SCA	95.38	91.08
Backbone	95.90	91.90
Backbone+ASPP	95.99	92.47
SSCA-Net	96.21	92.70

## Data Availability

The data used to support the findings of this study are available from the corresponding author upon request.
